# Phospholipid tail asymmetry allows cellular adaptation to anoxic environments

**DOI:** 10.1016/j.jbc.2023.105134

**Published:** 2023-08-09

**Authors:** Luca Panconi, Chris D. Lorenz, Robin C. May, Dylan M. Owen, Maria Makarova

**Affiliations:** 1 Institute of Immunology and immunotherapy, School of Mathematics and Centre of Membrane Proteins and Receptors (COMPARE), University of Birmingham, Birmingham, UK; 2 Department of Physics, King’s College London, London, UK; 3 Institute of Microbiology and Infection and School of Biosciences, University of Birmingham, Birmingham, UK; 4 School of Biosciences, Institute of Metabolism and Systems Research and Centre of Membrane Proteins and Receptors (COMPARE), University of Birmingham, Birmingham, UK

**Keywords:** lipids, membrane biophysics, cell adaptation, anoxia, acyl tails

## Abstract

Membrane biophysical properties are critical to cell fitness and depend on unsaturated phospholipid acyl tails. These can only be produced in aerobic environments since eukaryotic desaturases require molecular oxygen. This raises the question of how cells maintain bilayer properties in anoxic environments. Using advanced microscopy, molecular dynamics simulations, and lipidomics by mass spectrometry we demonstrated the existence of an alternative pathway to regulate membrane fluidity that exploits phospholipid acyl tail length asymmetry, replacing unsaturated species in the membrane lipidome. We show that the fission yeast, *Schizosaccharomyces japonicus*, which can grow in aerobic and anaerobic conditions, is capable of utilizing this strategy, whereas its sister species, the well-known model organism *Schizosaccharomyces pombe*, cannot. The incorporation of asymmetric-tailed phospholipids might be a general adaptation to hypoxic environmental niches.

The biophysical properties of cell membranes are critical for cell function and survival in varying environmental conditions. Cells use their metabolic capacity to generate lipidomes that optimize membrane properties, and thereby adapt to changing environmental niches. This is because it is membrane lipids that predominantly define membrane properties such as fluidity, viscosity, bending rigidity, and lateral diffusion (1). These properties in turn affect macroscale membrane activities such as curvature, fusion, fission, and the lateral distribution of membrane proteins (1, 2) Membrane properties are strongly defined by the hydrophobic core of the lipid bilayer, particularly by the phospholipid acyl tail composition. Fully saturated acyl tails form a tightly packed membrane with low-lateral diffusion and, in combination with sterols, form the so-called liquid-ordered phase. Alternatively, when acyl tails contain a double bond, a kink is introduced into the structure—which prevents tight lipid packing and forms a liquid-disordered phase with high lateral diffusion (3, 4, 5).

Desaturases are enzymes which introduce double bonds into acyl moieties in a process that is dependent on oxygen. Their di-iron center is activated by molecular oxygen to remove two hydrogens from the acyl chain and generate a double bond, and oxygen is also used as an electron acceptor in the desaturation reaction (6, 7), making oxygen a factor regulating membrane composition and properties. While some prokaryotic organisms can generate double bonds in the absence of oxygen during the synthesis of an acyl tail, this ability is not found in eukaryotes (8). The question therefore arises of how eukaryotic cells maintain membrane properties in anaerobic conditions when they are unable to produce unsaturated tail phospholipids.

The fission yeast *Schizosaccharomyces japonicus* is known to grow in both aerobic and anaerobic conditions and, compared to its well-studied relative *Schizosaccharomyces pombe*, has a demonstrated plasticity for generating diverse phospholipid acyl tail profiles. Recently it has been shown that the *S. japonicus* lipidome contains high quantities of asymmetric acyl chain phospholipids with 18:0 and 10:0 carbon atoms as the most predominant configuration (9). This asymmetry in acyl tails is not exclusive to *S. japonicus*, having also been identified in *Saccharomyces cerevisiae*, suggesting that these structural phospholipids could be more abundant than previously recognized (10). Our earlier work using artificial membrane systems has shown that these asymmetric phospholipids are able to replace unsaturated lipids to maintain the biophysical properties of simple, artificial membrane systems (11). We therefore hypothesize that the ability of *S. japonicus* to produce these asymmetric phospholipids may be behind its adaptation to anaerobic environments.

Here, we show that in the absence of oxygen, these asymmetric acyl tails fully replace unsaturated moieties and allow cells to maintain membrane properties to ensure cell fitness. We also observe changes in glycerophospholipid head groups and in sphingolipid precursors such as ceramides (Cer). Deletion of the membrane-bound desaturase *ole1 Δ9* resulted in a similar shift toward fully saturated, asymmetric lipids. At 37 °C, neither the lack of oxygen nor desaturase deletion affected cell fitness, suggesting that saturated, asymmetric lipids can substitute for their unsaturated counterparts. However, this compensation was only possible at physiologically high temperatures, since cells grown at 24 °C exhibited growth defects. Molecular dynamics (MD) simulations and advanced microscopy using environmentally sensitive probes traced this effect to an inability of asymmetric lipids to maintain membrane fluidity at these lower temperatures. Therefore, while asymmetric lipids may have evolved as a strategy for organisms to maintain membrane fluidity in the absence of the unsaturated lipids that molecular oxygen allows, this strategy is only partially successful.

## Results

### Lack of oxygen induces lipidome rewiring in *S. japonicus*

**Figure 1:**
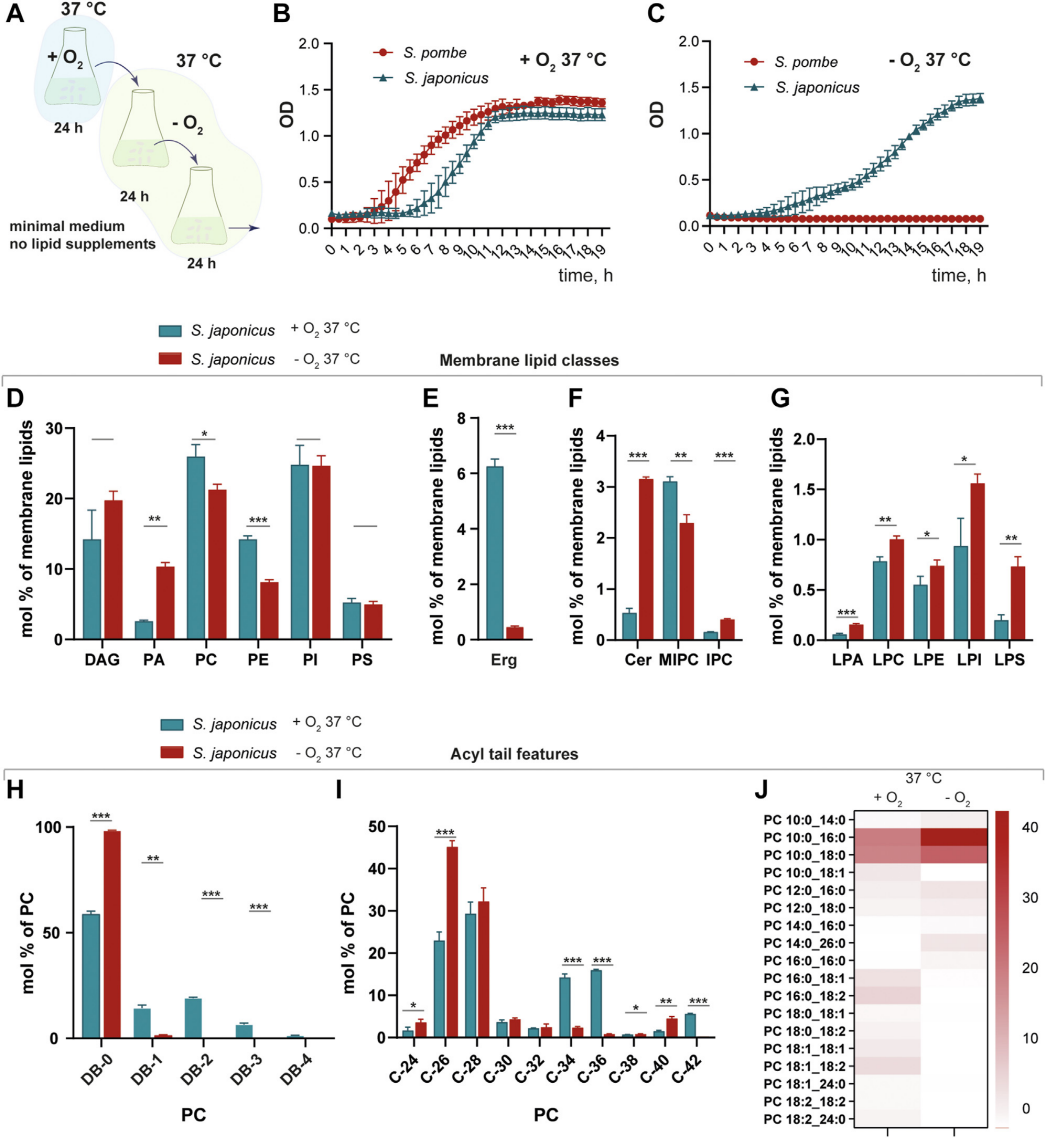
***Schizosaccharomyces japonicus* grown in anoxic conditions alter their membrane lipidome.***A*, scheme of growth experiment in the absence of oxygen; cells were grown in minimal medium conditions for 24 h in the presence of oxygen to mid-log growth phase then transferred to the anaerobic chamber and diluted with minimal medium without oxygen. Cells were allowed to grow to mid-log phase in an anaerobic chamber before growth and lipidomic experiments. *B*, growth curves of *S. japonicus* and *S. pombe* in minimal medium with oxygen measured as a change of absorbance (OD) at 600 nm. *C*, growth curves of *S. japonicus* and *S. pombe* in minimal medium without oxygen were measured as a change of OD. *D*, relative abundance of the main membrane phospholipid and glycerolipid classes presented as a molecular percentage of all identified membrane lipids in *S. japonicus* grown in the presence and absence of oxygen at 37 °C. *E*, relative abundance of ergosterol in *S. japonicus* grown in the presence and absence of oxygen at 37 °C. *F*, relative abundance of sphingolipids in *S. japonicus* grown in the presence and absence of oxygen at 37 °C. *G*, relative abundance of lysophospholipids in *S. japonicus* grown in the presence and absence of oxygen at 37 °C. *H*, relative abundance of double bonds in acyl tails within the PC class in *S. japonicus* grown in the presence and absence of oxygen at 37 °C. *I*, relative abundance of combined acyl-tail carbons within the PC class in *S. japonicus* grown in the presence and absence of oxygen. *J*, heat map showing molecular species composition within PC class in *S. japonicus* grown in normoxic and anoxic conditions. *Color bar* represents molecular percentage. PC, phosphatidylcholine.

To investigate how the membrane lipidome adapts to anaerobic growth in *S. japonicus*, we first assessed its growth pattern compared to *S. pombe*. To fully deplete lipids that had been synthesized in the presence of oxygen, we grew cells in an anaerobic cabinet for 48 h in a synthetic mineral medium and reinoculated cells into fresh oxygen-free medium every 24 h (Fig. 1*A*). After 48 h in anaerobic conditions, we measured cell growth and confirmed that *S. japonicus* growth rates were similar in both aerobic and anaerobic conditions (Fig. 1, *B* and *C*). In contrast, *S. pombe* showed complete growth suppression in anaerobic conditions (Fig. 1*C*).

To assess how *S. japonicus* adapts its lipid composition in the oxygen-free environment, we quantitatively analyzed around 600 unique lipid species *via* shotgun mass spectrometry (MS) in cells grown for at least 48 h anaerobically in minimal medium at 37 °C, in comparison to cells that had been grown aerobically (Fig. S1, *A*–*L* and Table S1). Adaptation to anaerobic growth dramatically remodeled membrane lipidome. We observed two main aspects of lipidome adaptation: lipid class (head group) changes and acyl tail remodeling. In glycerophospholipids, we observed an increase in phosphatidic acid (PA) and a decrease in two important phospholipid classes; phosphatdylcholine (PC), and phosphatidylethanolamine (PE) (Fig. 1*D*). Strikingly, prolonged growth in anaerobic conditions caused both complete depletion of esterified ergosterol and a significant decrease in ergosterol content (Fig. 1*E* and Table S1). Sphingolipids were also altered in anaerobic growth with an increased presence of Cers and inositolphosphoryl-ceramide (IPC) and decreased mannosylinositol-phosphorylceramide (MIPC) (Fig. 1*F*). In anaerobic conditions all classes of lysophospholipids were increased, suggesting major glycerophospholipid remodeling occurring during anaerobic growth in *S. japonicus* (Fig. 1*G*). Prolonged growth of *S. japonicus* in anaerobic conditions also caused significant remodeling in the lipid hydrophobic chain makeup, that is, length and saturation (Fig. 1, *H*–*J*). The lack of oxygen induced major rewiring in acyl chain composition in all identified classes of phospholipid, with unsaturated tails replaced by fully saturated counterparts (Fig. 1*H* - for PC, the distribution of lipid species in other classes is shown in Fig. S1, *F*–*I* and Table S2). This was accompanied by a decrease in the average acyl tail's length with lipid species that had a total of 34 or 36 carbon atoms in both tails being replaced by lipids mainly containing 26 carbon atoms (Fig. 1*J*). We also observed the decrease in acyl tail length in sphingolipids (Fig. S1, *J*–*L*).

Previously, it is been shown that the *S. japonicus* lipidome is naturally enriched in asymmetric acyl tail lipids with a total carbon length of 26 or 28 atoms and with individual tails 18:0/16:0 and 10:0 as the most predominant configuration (9). We confirmed that these 26-carbon tail lipid therefore represent the asymmetric 16:0,10:0 species and that they are significantly increased in anaerobic conditions (Fig. 1*J* for PC, the distribution of lipid species in other classes is shown in Fig. S1, *A*–*E*).

Overall, then, *S. japonicus* is able to grow normally in anaerobic conditions, whereas *S. pombe* is not. The lack of oxygen induces dramatic changes in the *S. japonicus* lipidome with a complete loss of acyl tail unsaturation and a dramatic increase in asymmetric phospholipids, primarily with saturated tails containing 16 and 10 carbons, respectively.

### *S. japonicus* upregulates asymmetric lipids specifically due to the absence of unsaturated acyl tails

**Figure 2:**
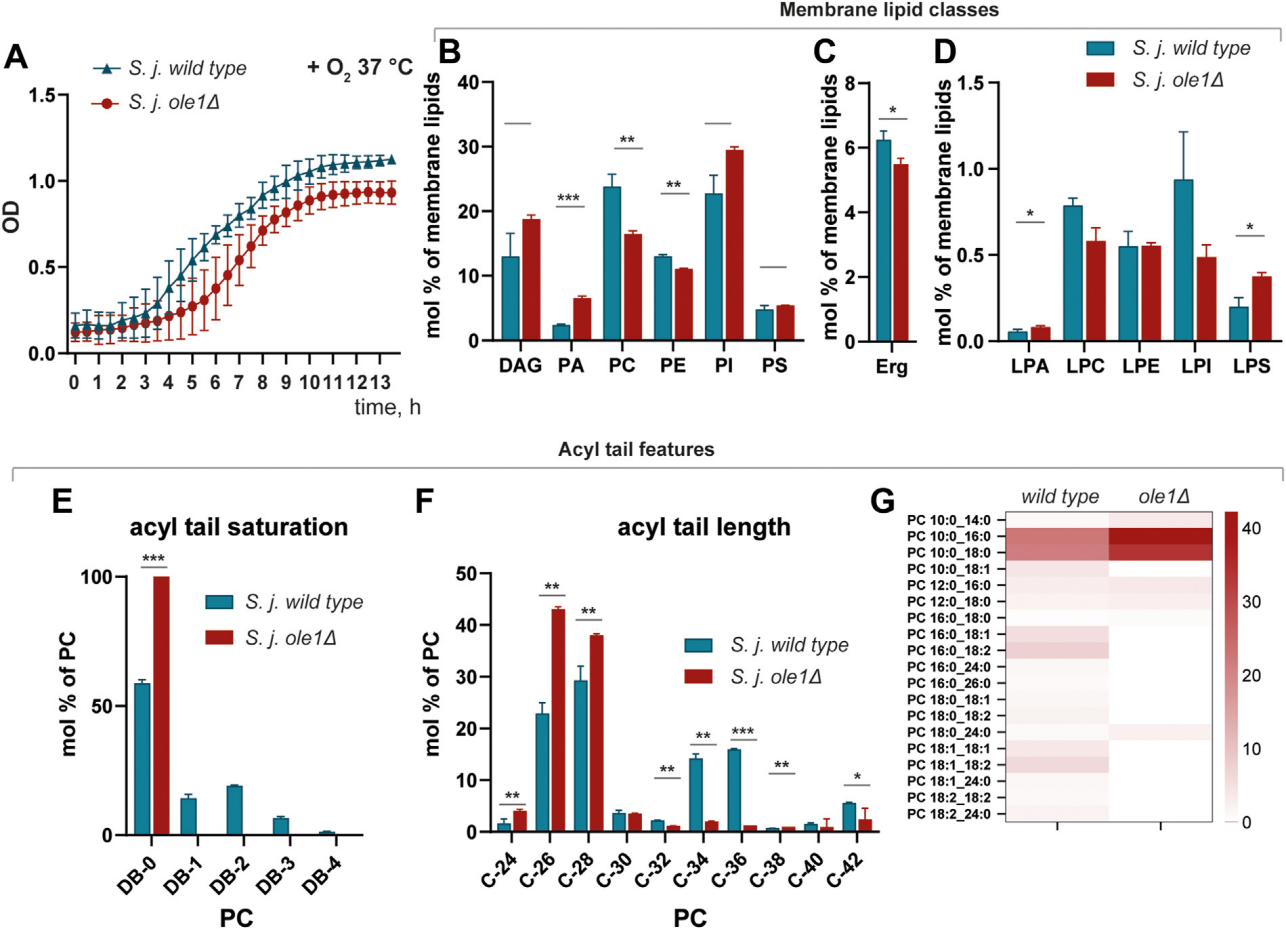
***Schizosaccharomyces japonicus* lacking desaturase *ole1* also generate saturated, asymmetric phospholipids.***A*, growth curves of *S. japonicus* WT and *S. japonicus ole1*Δ showing OD changes at 37 °C. *B*, relative abundance of the main membrane lipid classes in *S. japonicus* WT and *ole1*Δ were presented as molecular percentage of identified membrane lipids. *C*, relative abundance of ergosterol in *S. japonicus* WT and *ole1*Δ. *D*, relative abundance of lysophospholipids in *S. japonicus* WT and *ole1*Δ. *E*, relative abundance of double bonds in acyl tails within PC class in *S. japonicus* WT and *ole1*Δ. *F*, relative abundance of various carbon lengths in acyl tails in *S. japonicus* WT and *ole1*Δ. *G*, a heat map showing acyl tail composition within PC class in *S. japonicus* WT and *ole1*Δ. PC, phosphatidylcholine.

The main desaturase in fission yeast that introduces the double bond into acyl moieties is the Ole1 Δ9 desaturase, which has been shown to require molecular oxygen (12). We hypothesized that the increase in asymmetric lipids is a mechanism for the cell to compensate for the inability to synthesize unsaturated acyl tails in anaerobic conditions. To test this, we generated a mutant *S. japonicus* strain that lacks *ole1 Δ9* desaturase. A similar mutation in *S. pombe* or *S. cerevisiae* requires exogenous supplementation with oleic acid due to the poor growth of these mutants (13, 14, 15). In contrast, *S. japonicus* cells lacking *ole1* grow similarly to the WT strain in minimal medium conditions at 37 °C, relying solely on endogenous fatty acid synthesis (Fig. 2*A*).

To assess alterations to the membrane lipidome in the *ole1Δ* mutant compared to WT, we performed a similar lipidomic analysis where we compared WT *S. japonicus* cells to *ole1Δ* mutant cells. Quantitative analysis revealed similar changes as were identified in anaerobic conditions. First, as expected, unsaturated lipids were absent in the *ole1Δ* mutant, indicating that the deletion was successful in preventing the production of unsaturated phospholipids (Fig. S2, *F*–*I*). Second, PA was significantly increased and both PC and PE were decreased (Fig. 2*B*). Also, we observed some increase in the levels of two lysolipid classes—lysophosphatidate (LPA) and lysophosphatidylserine (LPS) (Fig. 2*D*). Third, and most interesting, the acyl tail length was decreased in a similar manner to that observed for WT cells grown in anaerobic conditions (Fig. 2, *E*–*G* for PC, the distribution of lipid species in other classes is shown in Fig. S2, *A*–*E* and Tables S3 and S4).

An important difference between the desaturase deletion mutant and cells grown in anaerobic conditions is that the ergosterol content in the mutant remained at a similar level as the WT cells (Fig. 2*C*). Ergosterol synthesis also requires oxygen (16), but was only mildly affected by *ole1* deletion. This implies the generation of asymmetric lipids is a compensation mechanism specifically for the absence of unsaturated lipids. It also shows that their presence in the bilayer is not a byproduct of any other metabolic changes that a lack of oxygen might have induced or a consequence of any nonspecific effects on cell physiology. We also did not observe remodeling of Cers as we did anaerobic conditions (Fig. S2, *J*–*L*).

### *S. japonicus* membrane properties can be maintained at 37 °C in the absence of Ole1 or oxygen

Cells regulate the lipid composition of membranes to accommodate certain biophysical properties that in turn facilitate membrane-associated cellular processes. Previously it has been shown that (a) *S. japonicus* membranes are more ordered than those of *S. pombe* and (b) in artificial model membranes, saturated asymmetric lipids can substitute for unsaturated lipids in maintaining membrane lipid order (17). We therefore hypothesized that *S. japonicus* membrane order would be maintained even in the absence of the unsaturated lipids. To investigate this, we used the environmentally sensitive probe di-4-ANEPPDHQ, which reports on membrane lipid order through changes in its fluorescence emission. This dye exhibits a red-shifted emission in more disordered membranes. The generalized polarization (GP) parameter, derived from the fluorescence measurements, ranges between +1 and −1, with higher values representing higher lipid order and packing (18).

**Figure 3:**
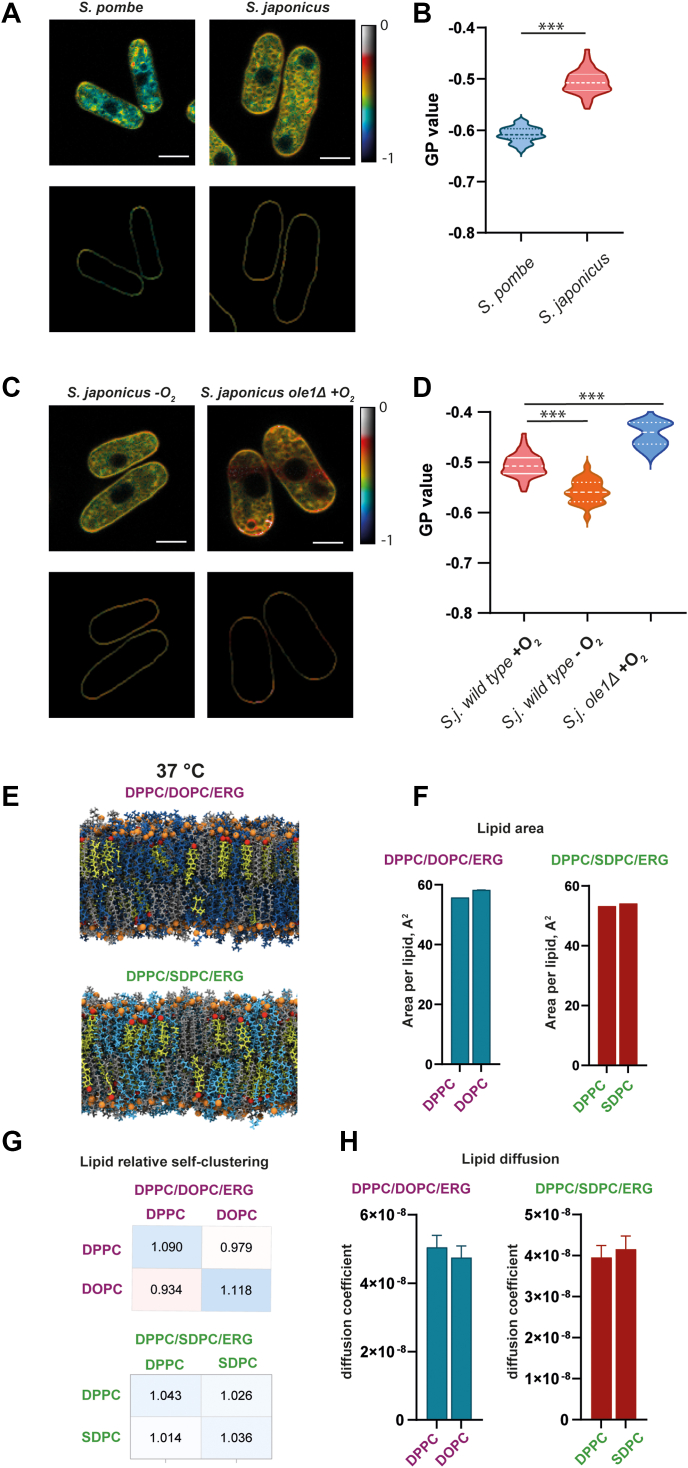
***Schizosaccharomyces japonicus* lacking desaturase ole1 maintains membrane properties at 37 °C.***A*, Pseudocolored generalized polarization (GP) images of *S. pombe* and *S. japonicus* grown in oxygen stained with polarity-sensitive dye di-4-ANEPPDHQ (*upper panel*) and cell outlines were used to quantify GP values of the plasma membrane (*lower panel*). The scale bar represents 5 μm. *B*, GP values quantified from *S. japonicus* (n = 53) and *S. pombe* (n = 101) were grown in aerobic setup cells. *C*, Pseudocolored GP images of *S. japonicus* grown in the absence of oxygen and *ole1*Δ in the presence of oxygen stained with polarity-sensitive dye di-4-ANEPPDHQ (*upper panel*) and cell outlines were used to quantify GP values of the plasma membrane (*lower panel*). The scale bar represents 5 μm. *D*, corresponding GP values of *S. japonicus* (n = 53) in aerobic condition, *S. japonicus* in anaerobic (n = 28), and *S. japonicus ole1*Δ (n = 31). *E*, snapshots of atomistic MD simulations of the two three-component bilayers: symmetric desaturated PC and asymmetric saturated PC. Biophysical properties of bilayers extracted from atomistic MD simulations (*F*–*H*). *H*, area per lipid, (*G*) lipid neighborhood, and (*H*) lipid diffusion coefficient. MD, molecular dynamics; PC, phosphatidylcholine.

Live-cell imaging indicated that the plasma membranes of *S. japonicus* cells growing aerobically at 37 °C are more ordered than those of *S. pombe* (Fig. 3, *A* and *B*). Both mechanisms for altering acyl tail composition in *S. japonicus* (genetically, through deletion of *ole1*, and conditionally, through growing cells in an oxygen-free environment) resulted in only small changes in lipid bilayer order (Fig. 3*C*). Deletion of *ole1* resulted in an increase in membrane order compared to the WT. This is an expected result of the lack of tail unsaturation and is consistent with previous results on artificial membranes (17). Anaerobically grown cells displayed lower membrane order than aerobically grown cells, possibly due to the decrease in ergosterol content. Ergosterol also requires oxygen for its synthesis, an effect which is obviously not present in the *ole1* deletion condition (Fig. 1*E*).

To assess the effect of a lack of unsaturated lipids on other membrane properties, we performed atomistic scale MD simulations on simple model membranes designed to exhibit phase separation, with compositions chosen to replicate those typically used in model membrane systems. Therefore, we simulated three-component lipid bilayers composed of either dipalmitoylphosphatidylcholine (DPPC, saturated lipid), dioleoylphosphatidylcholine (DOPC, unsaturated lipid), and ergosterol representing the control, WT condition, or DPPC (saturated lipid), 1-stearoyl-2-decanoyl-sn-glycero-3-phosphatidylcholine ([SDPC], saturated, asymmetric lipid), and ergosterol representing the absence of unsaturation, compensated for by asymmetry. The systems were allowed to equilibrate and simulations were run for a further 1 ms.

First, we extracted the number of each lipid species in the immediate vicinity of each lipid molecule and quantified the degree of enrichment or exclusion relative to what would be expected for randomly distributed lipids. In the simulations of the DPPC/DOPC/ergosterol membrane, we find that the DPPC lipids prefer to associate with other DPPC lipids and the DOPC lipids prefer to associate with other DOPC lipids; whereas in the DPPC/SDPC/ergosterol membrane, we find that the DPPC and SDPC lipids are more well mixed (Fig. 3*G*). However, this difference in the distribution of lipids within the membranes does not affect the other physical properties of the lipids we have measured (area per lipid [APL] and lipid diffusion) (Fig. 3, *F* and *H*). We observed difference in the way how acyl tails sn1 and sn2 of lipids overlap between leaflets, interdigitation of DPPC was higher in DPPC/DOPC/ergosterol membrane compared to the DPPC/SDPC/ergosterol (Fig. S3).

Overall, at 37 °C *S. japonicus* cells grow normally in anaerobic conditions (Fig. 1*C*) and in the absence of ole1 (Fig. 2*A*). While both of these conditions result in the loss of unsaturated lipids from the membrane, cells can largely maintain their membrane biophysical properties including membrane lipid order and lipid diffusion. As previously shown in artificial membranes therefore (17), saturated, asymmetric phospholipids are able to compensate for the loss of unsaturated lipids caused by either desaturase depletion or oxygen deprivation.

### Asymmetric lipids fail to compensate for their unsaturated counterparts at below 24 °C

**Figure 4:**
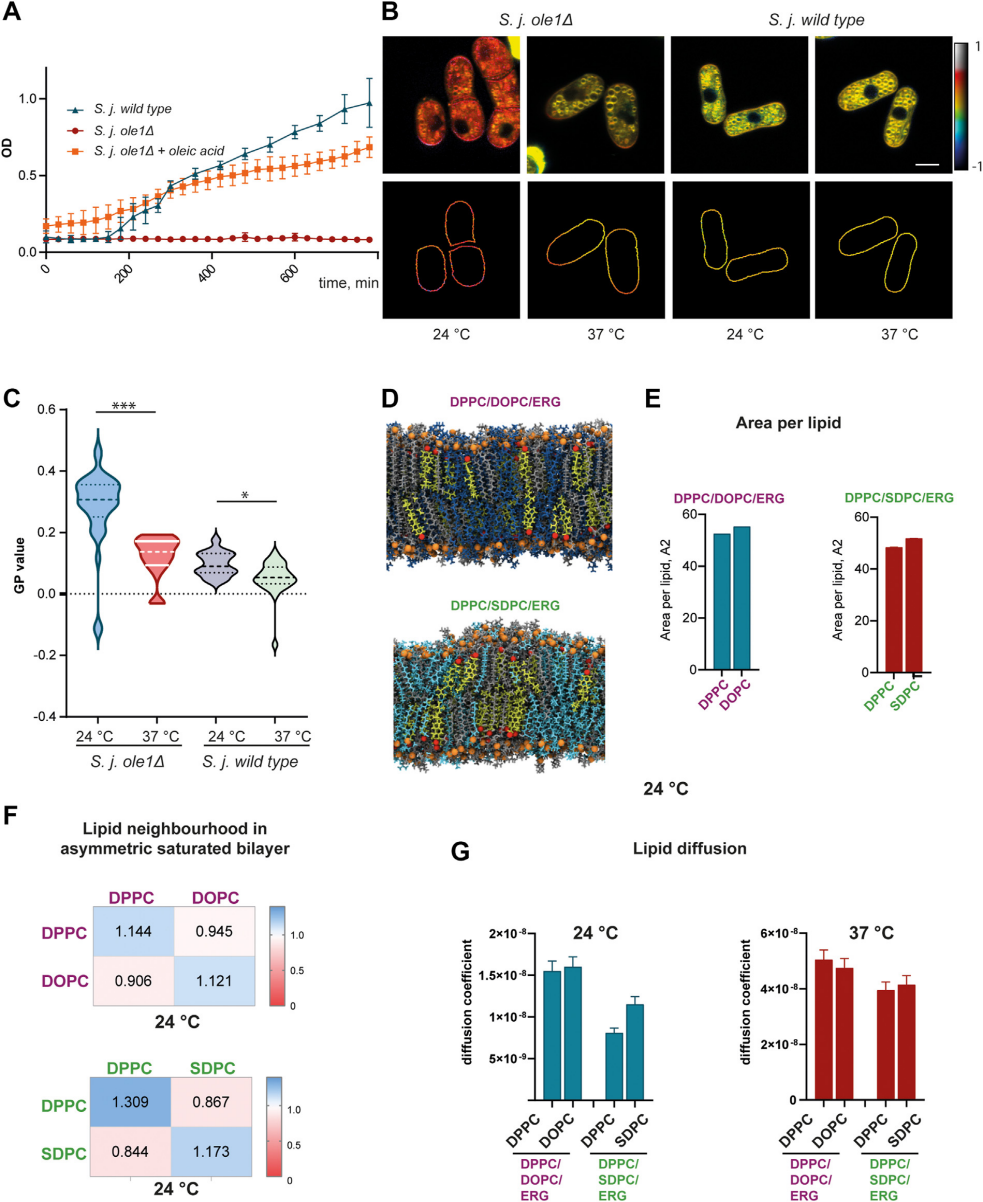
***Schizosaccharomyces japonicus* lacking desaturase *ole1* fails to sustain membrane properties at 24 °C.***A*, growth curves of *S. japonicus* WT and *ole1Δ*, *ole1Δ* supplemented with oleic acid at 24 °C. *B*, Pseudocolored GP images of *S. japonicus* WT and ole1Δ were grown at 24 °C and 37 °C and stained with polarity-sensitive dye di-4-ANEPPDHQ (*upper panel*) and cell outlines used to quantify GP values of the plasma membrane (*lower panel*). The scale bar represents 5 μm. *C*, GP values quantified from *S. japonicus ole1Δ* 24 °C (48 cells), *S. japonicus ole1Δ* 37 °C (58 cells), *S. japonicus WT* 24 °C (53 cells), and *S. japonicus WT* 37 °C (76 cells). *D*, snapshots of atomistic MD simulations of the two three-component bilayers: symmetric desaturated PC and asymmetric saturated PC. Biophysical properties of bilayers extracted from atomistic MD simulations (*E*–*G*). *E*, area per lipid, (*F*) lipid neighborhood, and (*G*) lipid diffusion coefficient. GP, generalized polarization; MD, molecular dynamics; PC, phosphatidylcholine.

At 37 °C asymmetric lipids, which do not require oxygen for their synthesis, are able to compensate for an absence of unsaturated lipids and closely maintain membrane properties. This raises the question of whether this is a more universal mechanism, and if so, why asymmetry is not well represented across diverse organisms. To answer this question, we investigated whether the *ole1Δ* mutant or anaerobically grown *S. japonicus* can grow at a similar range of temperatures as aerobically grown WT cells. Although we could not see any significant difference in growth at 37 °C (Figs. 1*C* and 2A), at 24 °C, the growth of *ole1*Δ mutant cells was significantly suppressed (Fig. 4*A*). To test whether this was specifically due to the lack of unsaturated lipids and not an unspecific temperature effect, we performed a rescue experiment. Ole1-deficient *S. japonicus* cells grown at 24 °C were supplemented with exogenous oleic acid (18:1). This rescued the growth phenotype, confirming the effect is specifically due to the lack of desaturation (Fig. 4*A*).

We hypothesized that the failure of ole1-deficient *S. japonicus* cells to grow at 24 °C might be the result of the failure of asymmetric lipids to provide sufficient compensation to maintain membrane properties at this lower temperature. GP values derived from fluorescence microscopy with environmentally sensitive dyes showed membrane order was increased at 24 °C compared to 37 °C for both WT and *ole1*Δ cells (Fig. 4, *B* and *C*), as expected (17). However, for WT cells the effect was extremely small and nonsignificant. In the *ole1*Δ mutant cells, however, GP values are dramatically increased. This indicated that the cells have failed to maintain membrane fluidity. MD simulations of three-component lipid bilayers performed at 24 °C show little changes in APL (Fig. 4*E*), but selfclustering increases dramatically (Fig. 4, *D* and *F*). This is particularly true in the system with saturated, asymmetric lipids (DPPC/SDPC/ergosterol), where there is a significant preference for the DPPC lipids to cluster with other DPPC lipids and SDPC lipids to cluster with other SDPC lipids at 24 °C, when at 37 °C, the two lipids were mixed. Most notably, diffusion coefficients only show small differences between 37 °C and 24 °C for bilayers containing unsaturated lipids but are decreased substantially when unsaturated lipids and replaced by saturated, asymmetric ones (Fig. 4*G*). Curiously, interdigitation and snorkeling of acyl tails in both lipid bilayers were similar between 37 °C and 24 °C temperatures (Figs. S3 and S4).

To conclude, while saturated, asymmetric lipids can substitute for unsaturated lipids at 37 °C, this is not the case at 24 °C. At the lower temperature, membrane biophysical properties are compromised, with a loss of fluidity and lipid diffusion. This in turn prevents cell growth, which can be rescued by exogenous supplementation with unsaturated fatty acids (Fig. 4*A*).

## Discussion

The fluidity of a bilayer is primarily determined by the relative abundance of unsaturated lipids, saturated lipids, and sterols, as well as the system’s temperature (19). Cells explore and optimise their membrane lipid composition to maintain bilayer physical properties in changing environments (20). We have previously shown that novel, saturated, asymmetric phospholipids can substitute for unsaturated lipids in the phase behavior of ternary artificial membrane systems (17). More specifically, a three-component bilayer formed from the asymmetric lipid SDPC together with the symmetric, saturated lipid DPPC and ergosterol will have similar phase and fluidity properties to bilayers composed of symmetric unsaturated lipids (DOPC), DPPC, and ergosterol. This may be because the asymmetry generates looser molecular crowding in the hydrophobic core of the bilayer, allowing a greater degree of acyl tail disorder. This leads to the hypothesis that asymmetric phospholipids might allow cells to maintain bilayer fluidity even in the absence of unsaturated lipids.

Here, we demonstrate that this is the case. In response to anoxic conditions, when the desaturase enzymes that produce unsaturated phospholipid tails cannot function, we found that *S. japonicus* produces an abundance of saturated but asymmetric tail phospholipids in response to anoxic conditions. These are capable of replacing symmetric unsaturated lipids in maintaining bilayer biophysical properties. Deletion of the main desaturase *ole1* demonstrated a similar response showing that asymmetric lipids are a more general adaptation to loss of acyl tail desaturation, rather than lack of oxygen *per se*. This adaptation allows *S. japonicus* to survive and proliferate in anoxic conditions provided high temperature is maintained. Below 24 °C, asymmetric lipids are unable to maintain bilayer fluidity in the same way unsaturated lipids can and growth is curtailed. Conversely, *S. japonicus’* sister species, the well-known model organism *S. pombe*, does not produce an abundance of asymmetric lipids and is incapable of growth in anoxic conditions.

The generation of asymmetric lipids to substitute for desaturation in order to maintain membrane fluidity may be a more general adaptation. *S. japonicus* may be adapted to natural hypoxic environmental niches, particularly those at higher temperatures. These might include, for example, compost, deep ocean crusts, or the digestive tracts of animals. Other organisms that also inhabit these conditions in nature may have developed adaptations to compensate for the difficulty in manufacturing unsaturated membrane lipids. Such information is limited, however, primarily because the MS methods to reliably detect asymmetric phospholipids have only recently been widely deployed.

How do asymmetric lipids affect cellular physiology and function? The physical properties of membranes formed from asymmetric lipids do differ from those formed from their symmetric counterparts. This is likely to affect process that require extensive membrane remodeling and require fine-tuning of membrane properties for their execution. Among those are vesicle formation and trafficking and the remodeling of the nuclear envelope during mitosis. Indeed, we know that *S. pombe* undergoes close mitosis that is characteristic of unicellular fungal species, whereas *S. japonicus* undergoes semiopen mitosis (21). We speculate that these physiological differences might be the result of altered membrane lipidomes are hence different membrane material properties.

How generalizable is this adaptation to hypoxia and is the exploitation of asymmetric lipids a monophyletic trait? The medium-chain fatty acids (MCFAs) that are required for the synthesis of asymmetric lipids have been observed to be dramatically increased in the species *Mucor rouxii* from the division mucoromycota (compared to the division Ascomycota for *S. pombe* and *S. japonicus*) (22). Asymmetric lipids themselves have been detected in *S. cerevisiae* under rich medium conditions (10), and there are numerous reports of changes to membrane lipidomes in a variety of hypoxic conditions, for example, within the tumor microenvironment or in deep sea marine organisms (23, 24, 25). Therefore, we believe that the exploitation of asymmetric lipids as an adaptation to hypoxia may have arisen multiple times in evolutionary history or might be a dormant feature of eukaryotic cells more generally.

By what mechanism can asymmetric phospholipid synthesis be upregulated? A key requirement is the manufacture of MCFAs (*e.g.*, C10) to be incorporated into phospholipids. Our previous work has shown that *S. japonicus* is capable of producing MCFAs, most likely through the adaptation of its fatty acid synthase (FAS) enzymes. Purified *S. japonicus* FAS was able to produce MCFAs *in vitro*, whereas *S. pombe* FAS could not (9). This might explain the capability of *S. japonicus* in producing MCFAs and hence asymmetric lipids, a capability lacking in *S. pombe*. In order to regulate the length profile of fatty acids produced by FAS, we hypothesize that cells may alter the ratio of acetyl-CoA and malonyl-CoA, which are responsible for fatty acid initialization and lengthening, respectively. Another possibility is that thioesterases, specifically thioesterase II have been shown to shift the product specificity of FAS from predominantly long to mainly MCFAs (26). The full fatty acid length profiles produced by any cell, therefore, is bounded genetically (through FAS) but also modified metabolically, for example, in response to environmental cues.

This work mainly explores the acyl tail makeup of glycerophospholipids, but we also found that in anaerobic conditions, there are other changes to the membrane lipidome of *S. japonicus*. For example, we observed changes in the lipid head groups. Primarily, we note a large increase in the proportion of PA and a smaller increase in diacylglycerol (DAG). Conversely, PE and PC are decreased. We speculate that this is a consequence of the cells reducing phospholipid synthesis with a resultant buildup of phospholipid precursor molecules (PA and DAG). This might be a reduction in the activity of the enzymes CDS1 or PAH1 in the lipid metabolic pathways (27, 28). Alternatively, remodeling of PC to PA could be due to the altered phospholipase D activity (29). A similar pattern might be evident for sphingolipids because we observe a dramatic increase in the prevalence of Cer in the hypoxic condition, possibly implicating the enzymes AUR1 and KEI1—which convert Cer into IPCs and CSG1—and 2 and CSH1—which convert IPCs into MIPCs (30, 31). Finally, storage lipids—triacylglycerol (TAGs)–were decreased in both anaerobic and *ole1* mutant conditions, possibly suggesting their consumption as a pool for acyl tail remodeling (32).

Despite the insights provided here, several important questions remain unanswered. For example, sterols, of which ergosterol is the most common in fungi also require molecular oxygen for their synthesis (33). Since sterols are also important for maintaining membrane fluidity, it is likely that *S. japonicus* may also seek to compensate for the lack of membrane sterols. We hypothesize that hopanoids might represent a candidate substitute for sterols (34). We also note that our lipidomic studies are conducted in whole-cell lysates, whereas GP measurements are from the plasma membrane only. This generates additional uncertainty which would require subcellular fractionation to help resolve. It is also unclear what property of the membrane cells sense in order to respond through changes in their lipidome. Our *ole1* deletion experiment shows that they are not sensing the lack of oxygen directly, or any other metabolic changes that might be the result of anoxia. However, they may be sensing the absence of acyl tail desaturation or a property of the bilayer, for example, its lipid packing density or thickness (35).

Overall, we have detailed how the ability to synthesize saturated, asymmetric phospholipids allow *S. japonicus* to thrive in anoxic conditions by allowing the organism to maintain the biophysical properties of its membranes. We hypothesize that this might be a more general mechanism for cells and organisms that inhabit hypoxic environmental niches. In general, the adaptation of the membrane lipidome to changing environmental conditions and how lipidomes are genetically and metabolically defined remain poorly understood.

## Experimental procedures

### Yeast strains and plasmids

Yeast strains and primers used in this study are listed in Table S1. The deletion of *ole1* gene in *S. japonicus* was performed using a plasmid-based homologous recombination strategy. For that 3′UTR fragment and 5′UTR fragment of *ole1* gene were gene synthesized and cloned in the plasmid with kanR cassette. The plasmid was linearized with SmaI and transformed into WT *S. japonicus* cells as described previously (36). Cells that were successfully transformed were selected on G418 (final concentration 60 μg/ml) for 3 days at 37 °C. The KO mutant was verified by PCR with the primers listed in Table S1.

### Cell growth and media

For genetic modification and cell propagation, *S. japonicus* and *S. pombe* cells were grown in yeast extract supplemented medium described in (37, 38). For lipidomic and growth experiments cells were grown Edinburgh minimal medium. For anaerobic experiments, cells were grown initially in aerobic conditions shaking at 170 rpm to mid-log growth phase until ∼0.6 OD then shifted to anaerobic cabinet Don Whitley Scientific A35. Cells were diluted to 0.01 OD in a minimal medium that would lack oxygen and grown at 37 °C for 24 h. The next day, cells were diluted again to 0.01 OD without exposure to atmospheric oxygen and grown for another 24 h before any growth, lipidomic, and lipid order measurements. This was done to ensure that lipids generated in the presence of oxygen would be used up by the time when samples for lipidomic analysis or growth experiments were collected.

For growth experiments cell absorbance was measured in aerobic conditions using FLUOstar Omega plate reader at the indicated temperatures in 96-well plates, the measurements were taken every 30 min. For growth measurements in anaerobic conditions, a portable Cerillo Stratus plate reader was used, and cell densities were measured in six technical replicates and three biological independent replicates in 96-well plates.

### Lipid extraction for MS lipidomics

MS-based lipid analysis was performed by Lipotype GmbH as described (39, 40). Lipids were extracted using a two-step chloroform/methanol procedure (40). Samples were spiked with internal lipid standard mixture containing: CDP-DAG 17:0/18:1, cardiolipin 14:0/14:0/14:0/14:0, Cer 18:1;2/17:0, DAG 17:0/17:0, LPA 17:0, lysophosphatidylcholine 12:0, lysophosphatidylethanolamine 17:1, lysophosphatidylinositol 17:1, LPS17:1, PA 17:0/14:1, PC 17:0/14:1, PE 17:0/14:1, phosphatidylglycerol 17:0/14:1, phosphatidylinositol 17:0/14:1, phosphatidylserine 17:0/14:1, ergosterol ester 13:0 , TAG 17:0/17:0/17:0, stigmastatrienol, IPC 44:0;2, MIPC 44:0;2, and mannosyl-di-(inositolphosphoryl)ceramide 44:0;2. After extraction, the organic phase was transferred to an infusion plate and dried in a speed vacuum concentrator. First step dry extract was resuspended in 7.5 mM ammonium acetate in chloroform/methanol/propanol (1:2:4, V:V:V) and second step dry extract in 33% ethanol solution of methylamine in chloroform/methanol (0.003:5:1; V:V:V). All liquid handling steps were performed using Hamilton Robotics STARlet robotic platform with the Anti Droplet Control feature for organic solvents pipetting.

### MS data acquisition

Samples were analyzed by direct infusion on a QExactive mass spectrometer (Thermo Fisher Scientific) equipped with a TriVersa NanoMate ion source (Advion Biosciences). Samples were analyzed in both positive and negative ion modes with a resolution of Rm/z = 200 = 280,000 for MS and Rm/z = 200 = 17,500 for MS/MS (mass spectrometry/mass spectrometry) experiments, in a single acquisition. MS/MS was triggered by an inclusion list encompassing corresponding MS mass ranges scanned in 1 Da increments (41). Both MS and MS/MS data were combined to monitor ergosterol ester, DAG, and TAG ions as ammonium adducts; PC as an acetate adduct; and cardiolipin, PA, PE, phosphatidylglycerol, phosphatidylinositol, and phosphatidylserine as deprotonated anions. MS only was used to monitor LPA, lysophosphatidylethanolamine, lysophosphatidylinositol, LPS, IPC, MIPC, mannosyl-di-(inositolphosphoryl)ceramide as deprotonated anions; Cer and lysophosphatidylcholine as acetate adducts and ergosterol as protonated ion of an acetylated derivative (42).

### MS data analysis and postprocessing

Data were analyzed with in-house developed lipid identification software based on LipidXplorer (https://lifs-tools.org/lipidxplorer.html) (43). Data postprocessing and normalization were performed using an in-house developed data management system. Only lipid identifications with a signal-to-noise ratio >5, and a signal intensity 5-fold higher than in corresponding blank samples were considered for further data analysis.

### Measurements of membrane lipid order *in vivo*

For imaging, cells were grown in similar conditions for lipidomic analysis and growth experiments, in minimal medium in the presence or absence of oxygen at indicated temperatures overnight, and the following day they were diluted to OD 0.2 in the fresh medium containing 5 μM di-4-ANEPPDHQ. Cells were incubated for 2 h and then transferred into a glass-bottomed microscope dish. Imaging for Figure 3 was performed on a Zeiss LSM 780 confocal microscope equipped with a 32 element GaAsP Quasar detector. A 488 nm laser was selected for fluorescence excitation of di-4-ANEPPDHQ. The detection windows were set to 510 to 580 nm and 620 to 750 nm. Images from three independent experiments were used for analysis. The setting on the microscope were kept the same between independent experiments. For images in Figure 4 a Zeiss LSM 880 confocal microscope equipped with a 32 element GaAsP Quasar detector with Airyscan was used to obtain images, the settings between sets of experiments were not maintained hence different GP values were obtained between the experiments.

### Image analysis

Image preprocessing was undertaken in Fiji image manipulation software version 2.5.0 (https://fiji.sc/), available under GNU General Public License version 3.0. To increase cell visibility, a copy of each image was created and the auto-contrast plugin was applied. Cell segmentation was then undertaken using the TOBLERONE software package (44) written in the R programming language version 4.2.0, employed within the RStudio integrated development environment (https://github.com/lucapanconi/toblerone). Segmentation was achieved by varying the input parameter until the expected number of cells was identified in each image. For each segmented cell, the oriented boundary corresponding to the plasma membrane was then extracted. Generalized polarization values were calculated on the original images using built-in functions from the TOBLERONE package. Further analysis was undertaken using built-in statistical techniques in R.

### MD simulations

Two different lipid bilayers were simulated in order to investigate the effect of lipid molecules with asymmetric tails on the properties of tertiary bilayers where one bilayer consisted of 35% DPPC, 35% DOPC, and 30% ergosterol and the other consisted of 35% DPPC, 35% SDPC and 30% ergosterol. Each membrane was built with 200 lipids in each leaflet using the CHARMM-GUI membrane builder (45, 46). Each system also included 40 water molecules per lipid molecule as well as 0.15 mM NaCl. The two bilayers were each studied at two different temperatures, 295 K and 310 K. We performed two replica simulations for each bilayer at each temperature.

Each membrane was minimized and then equilibrated the desired temperature (either 295 K or 310 K) and a pressure of 1 bar, following the simulation protocol prescribed by CHARMM-GUI (47). After equilibrating each system, a production simulation was carried out for 1 μs at the desired temperature and a pressure of 1 bar. The temperature was controlled by a Nosé–Hoover thermostat and a Parrinello–Rahman barostat was used to control pressure.

All simulations were run using the GROMACS 2020 simulation package (48) and the CHARMM36 forcefield was used to model the interactions of the lipid molecules and the ions ((49), while the water molecules were modeled using CHARMM TIP3P (50). We used the same model for the SDPC lipid, which was generated by truncating the sn-2 tail of the CHARMM36 DSPC model after the 10th carbon, that we used in our previous study of asymmetric lipid membranes (17). LINCS constraints were used on the hydrogen-containing bonds in order to allow us to use 2 fs timesteps within the production simulations. The last 100 ns of production simulation was used for analysis.

The bilayer properties were characterized by the APL, bilayer thickness, and lipid order parameter (S_CD_) of the lipids within the bilayers. All analysis was conducted using in-house generated python scripts, which used MDAnalysis (51, 52) or LiPyphilic (53).

A 2D Voronoi tessellation of atomic positions in each leaflet was performed to determine the APL of each component of the bilayer, using the C21 C2 and C31 atoms as seeds for the PC lipids, and O3 atoms for the ergosterol (atoms are given by CHARMM atom names). We calculate the mean APL of each species at each frame, and calculate the standard deviation over time. The lipid order parameter is a measure of the conformational flexibility of acyl chains in a bilayer, and is given by:

$$S_{CD}=\left|\frac{1}{2}\;\langle 3\;\cos^{2}\left(\theta \right)-1\;\rangle \right|$$

where θ is the angle between the bilayer normal and the carbon-hydrogen vector of a carbon atom in an acyl tail, and the average is taken over time and over all molecules of a given species within the membrane. The S_CD_ was calculated for each lipid species as a function of carbon atom position along an acyl chain. Smaller values of S_CD_ indicate a more disordered acyl chain. To calculate the bilayer thickness, we constructed a lateral 6 × 6 grid of the membrane, calculated the mean phosphate–phosphate distance in each grid point, then averaged over the grid. This better accounts for a rough membrane surface, such as with DPPC in the ripple phase. We report the SD of the mean membrane thickness over time.

We calculated the lateral mean–squared displacement (MSD) of each species using the C2 and O3 atoms of the PC lipids and ergosterol, respectively, over the final 300 ns of each simulation. Before calculating the MSD of each lipid, we first removed the lateral center of mass motion of the bilayer. We then calculated the lateral diffusion coefficient (*D*) for each lipid using the Einstein relation:

$$D=\frac{1}{2d}\lim_{t\to \infty }\frac{\left\langle r_{t}-r_{t0}\right\rangle }{t}$$

where *d* is the system dimension, *r* is the coordinate of the C2 or O3 atom at a given time, *t*, from a time origin, *t*_*0*_. We used the period from 100 ns to 250 ns of the MSD curve to calculate *D* for each individual lipid. We then calculated the average and standard error of *D*.

## Data availability

The full dataset is available from the corresponding author upon reasonable request. All strains, custom-reagents, and raw data are freely available on request.

## Supporting information

This article contains supporting information.

## Conflict of interest

The authors declare that they have no conflicts of interest with the contents of this article.
