# Supplementary material for: Phospholipid tail asymmetry allows cellular adaptation to anoxic environments
Source: J Biol Chem. 2023 Aug 9;299(9):105134. doi: 10.1016/j.jbc.2023.105134 (PMC10482748; doi:10.1016/j.jbc.2023.105134)
Supplement: Supporting Figures S1–S4 [file mmc1.docx]

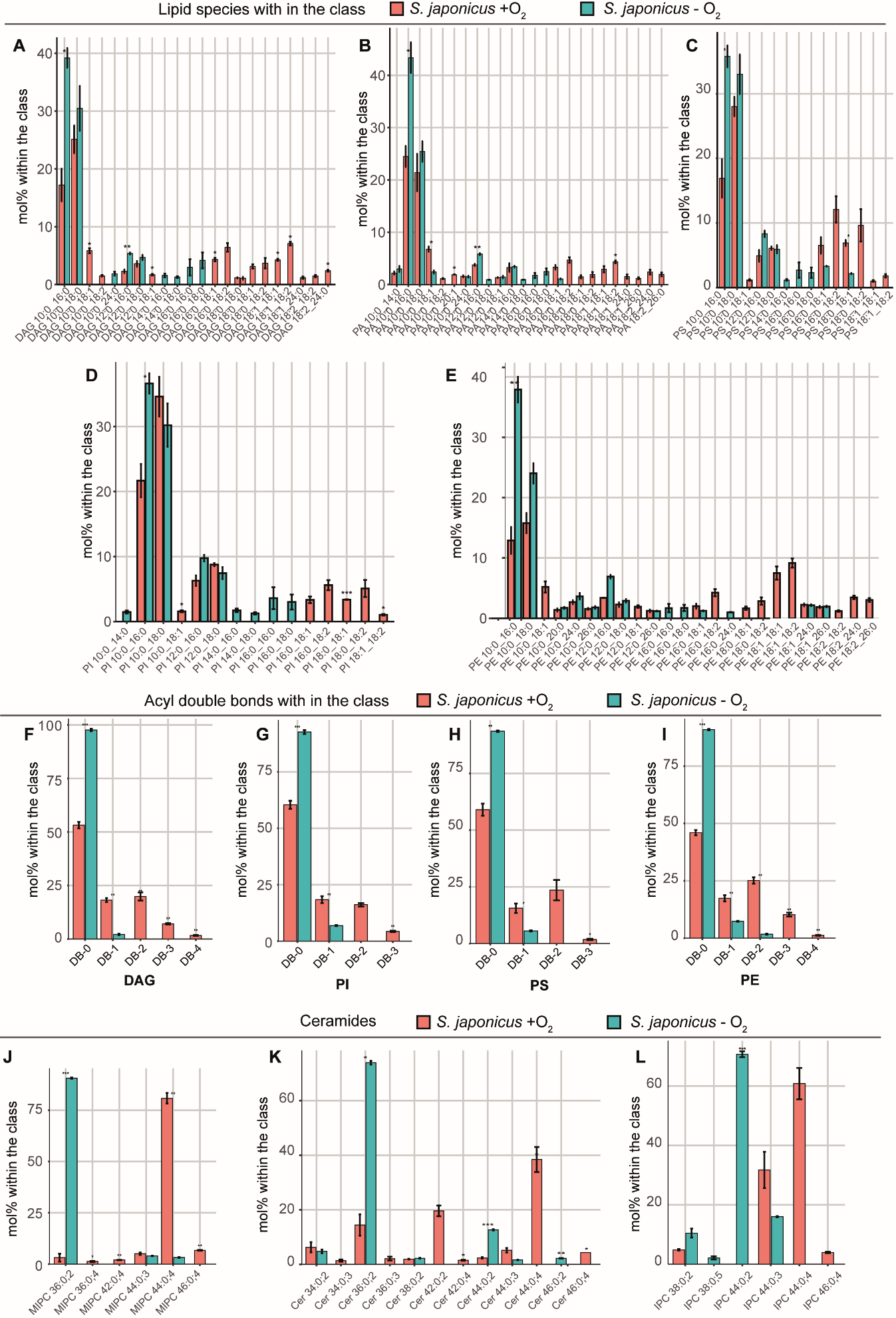


**Fig. S1.** **Quantitative lipidomic results for *S. japonicus* grown in normoxic and anoxic conditions** (A-E) Relative abundance of molecular lipid species within DAG (diacylglycerol), PA (phosphatidic acid), PE (phosphatidylethanolamine), PI (phosphatidylinositol) and PS (phosphatidylserine) classes in *S. japonicus* grown in normoxic and anoxic conditions. (F-I) Relative abundance of double bonds in acyl tails within DAG, PI, PS and PE classes in *S. japonicus* grown in normoxic and anoxic conditions. (J-L) Relative abundance of molecular lipid species within MIPC, Ceramides and IPC. The number after the semi-colon indicates the total hydroxy group number in corresponding molecular specie. Presented are the mean values of three biological replicates and SD values. T-test was used to indicate significant differences between two conditions.


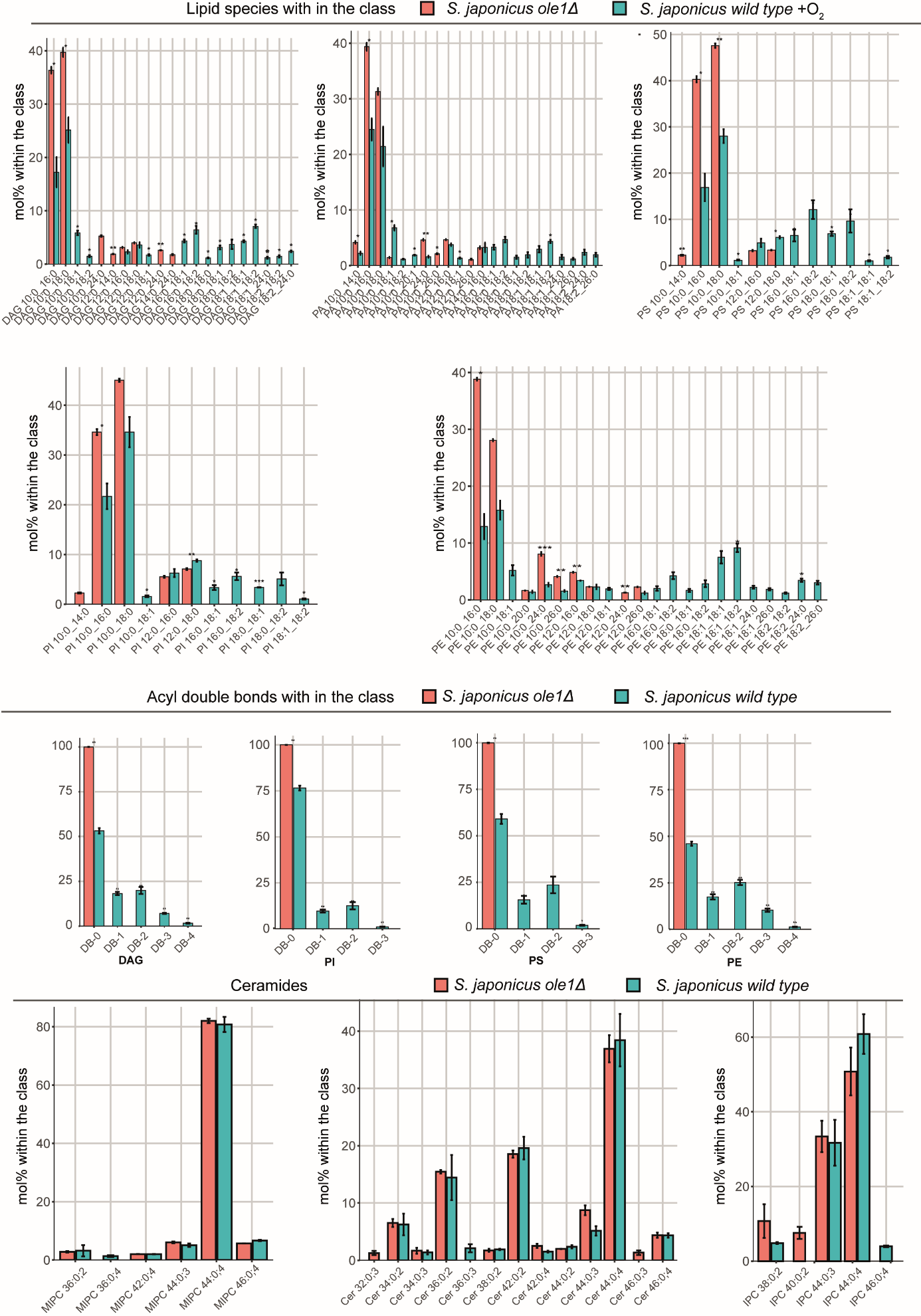


**Fig. S2. Quantitative lipidomic results for *S. japonicus* *ole1Δ* and *S. japonicus* wild type*.*** (A-E) Relative abundance of molecular lipid species within DAG (diacylglycerol), PA (phosphatidic acid), PE (phosphatidylethanolamine), PI (phosphatidylinositol) and PS (phosphatidylserine) classes in *S. japonicus* *ole1Δ* and *S. japonicus* wild type. (F-I) Relative abundance of double bonds in acyl tails within DAG, PI, PS and PE classes in *S. japonicus* *ole1Δ* and *S. japonicus* wild type. (J-L) Relative abundance of molecular lipid species within MIPC, Ceramides and IPC in *S. japonicus* *ole1Δ* and *S. japonicus* wild type. The number after the semi-colon indicates the total hydroxy group number in the corresponding molecular specie. Presented are the mean values of three biological replicates and SD values. T-test was used to indicate significant differences between two conditions.


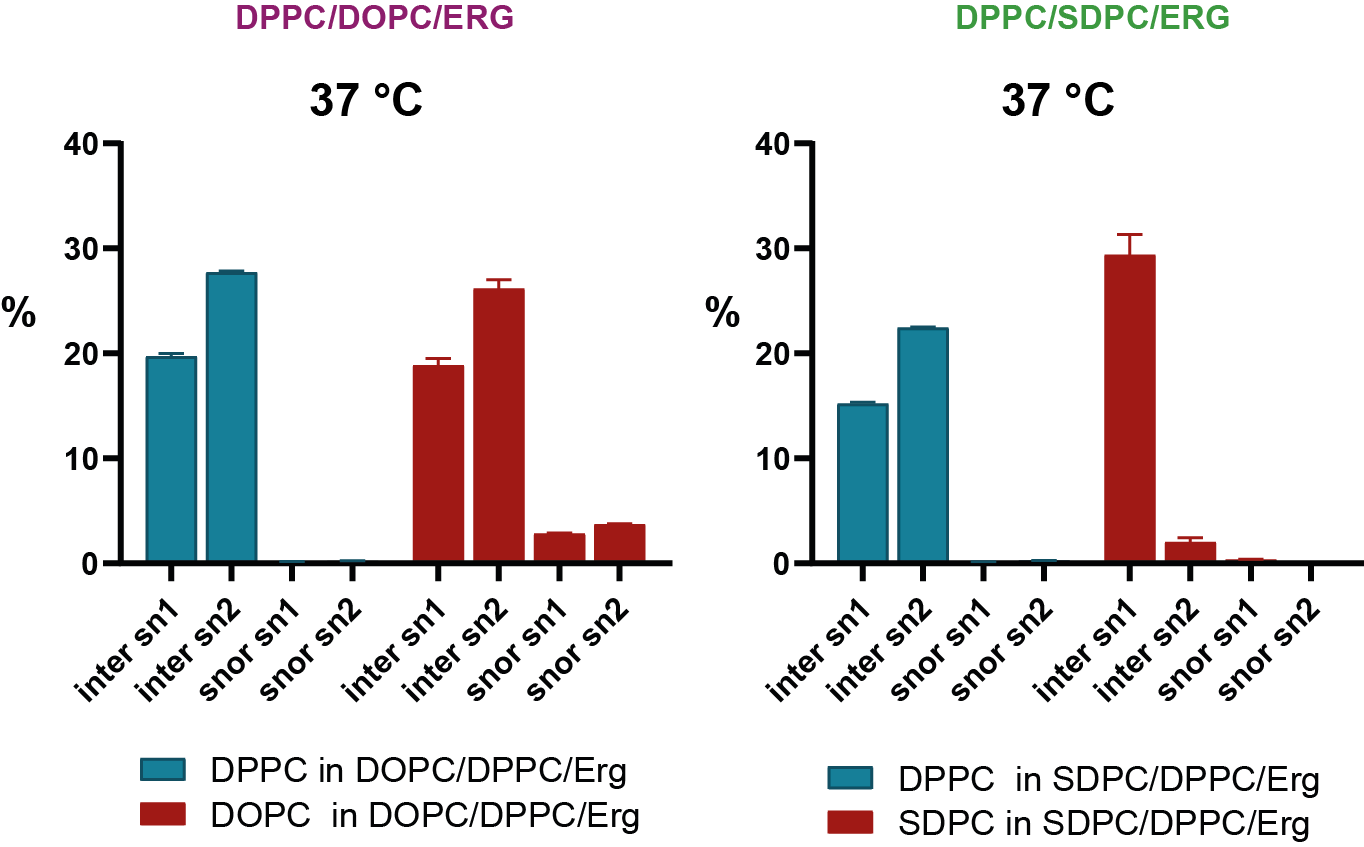


Fig. S3. Percentage of the terminal carbons in each sn acyl chain that interdigitate or snorkel at 37°C in simulated lipid bilayers composed of either DPPC/DOPC/Erg or DPPC/SDPC/Erg.


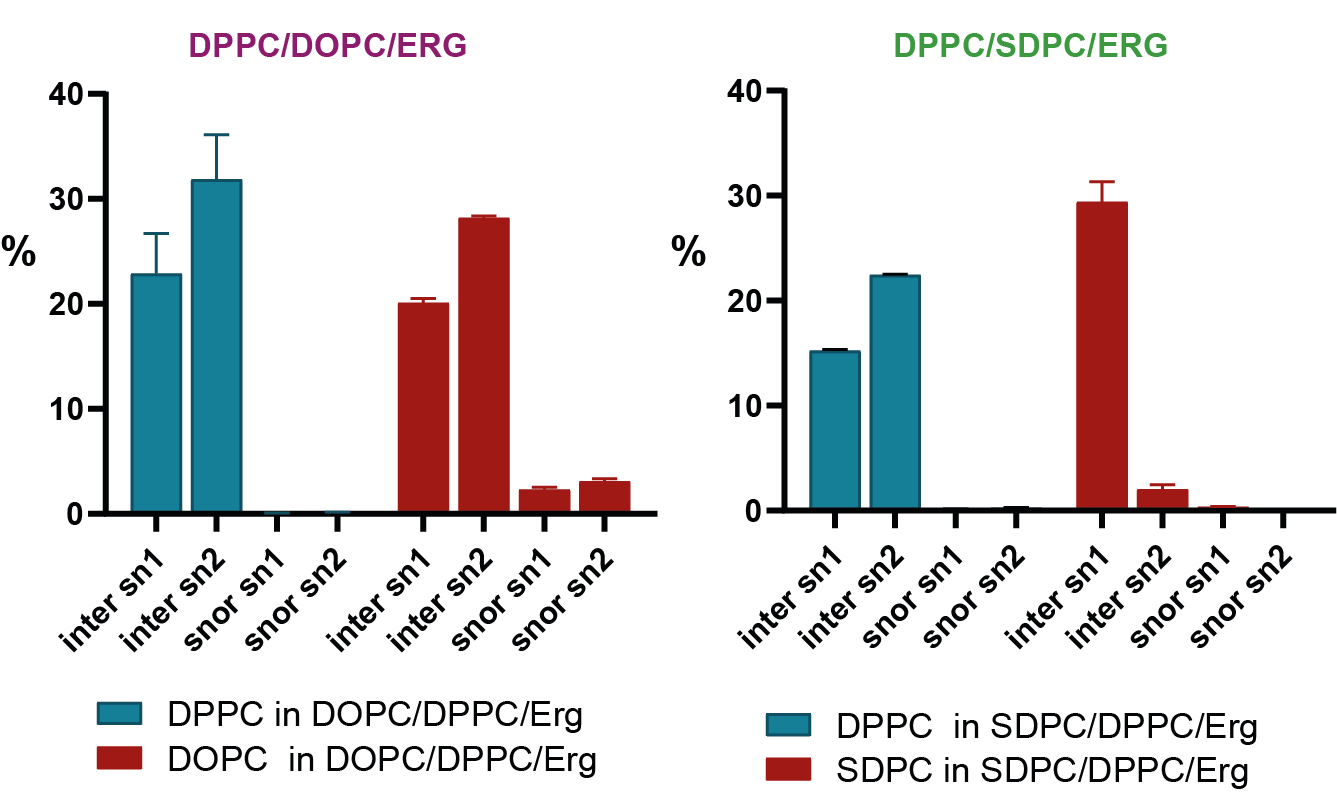


Fig. S4. Percentage of the terminal carbons in each of the sn acyl chains that interdigitate or snorkel at 24°C in simulated lipid bilayers composed of either DPPC/DOPC/Erg or DPPC/SDPC/Erg.

Data S1. (separate file)

Quantitative lipidomic data
